# A Case of Curative Resection by Minimally Invasive Surgery in Two Stages Using the Hormonal Mapping Test for Pancreatic Head ACTHoma with Multiple Liver Metastases

**DOI:** 10.70352/scrj.cr.25-0542

**Published:** 2025-12-20

**Authors:** Shohei Motohashi, Daisuke Asano, Satoshi Matsui, Yoshiya Ishikawa, Hiroki Ueda, Keiichi Akahoshi, Eriko Katsuta, Daisuke Ban

**Affiliations:** Department of Hepatobiliary and Pancreatic Surgery, Graduate School of Medicine, Institute of Science Tokyo, Tokyo, Japan

**Keywords:** pancreatic neuroendocrine neoplasm, ACTH-producing tumor, SASI test, SACI test, hormonal mapping

## Abstract

**INTRODUCTION:**

Symptom control is sometimes as important as tumor burden control in patients with functional neuroendocrine tumors (NETs). The selective arterial calcium injection (SACI) test is widely used to localize functional NETs, particularly insulinomas and gastrinomas; however, its utility in adrenocorticotropic hormone (ACTH)-producing tumors (ACTHomas) remains unclear. We present a case of a pancreatic head ACTHoma with synchronous liver metastases in which the SACI test with venous sampling (“hormonal mapping”) enabled a safe, staged curative resection.

**CASE PRESENTATION:**

A 42-year-old woman presented with features of Cushing’s syndrome, including edema and weight gain. CT revealed a pancreatic head tumor with multiple liver metastases. Liver biopsy confirmed the diagnosis of NET, suggesting an ACTHoma. Hormonal mapping demonstrated that liver metastases, rather than the pancreatic tumor, were the predominant sources of ACTH secretion. To achieve hormonal control, we prioritized laparoscopic liver resection, followed by laparoscopic pancreatoduodenectomy three months later after confirming the absence of new metastatic lesions. At 21 months postoperatively, the patient remains recurrence-free.

**CONCLUSIONS:**

Hormonal mapping was effective in identifying hormone-producing lesions and guiding surgical strategies for ACTHomas with liver metastases.

## Abbreviations

ACTH
: adrenocorticotropic hormone
ACTHoma
: ACTH-producing neuroendocrine tumor
DGE
: delayed gastric emptying
GDA
: gastroduodenal artery
IVC
: inferior vena cava
LHA
: left hepatic artery
LHV
: left hepatic vein
MHV
: middle hepatic vein
NELM
: neuroendocrine liver metastases
NET
: neuroendocrine tumor
PanNET
: pancreatic neuroendocrine tumor
PD
: pancreatoduodenectomy
PDAC
: pancreatic ductal adenocarcinoma
RHA
: right hepatic artery
RHV
: right hepatic vein
SACI test
: selective arterial calcium injection test
SpA
: splenic artery
SSA
: somatostatin analog
TACE
: transcatheter arterial chemoembolization

## INTRODUCTION

Pancreatic neuroendocrine tumors (PanNETs) are relatively rare neoplasms arising from the neuroendocrine cells of the pancreas and are generally less aggressive than pancreatic ductal adenocarcinomas (PDAC). The 5-year overall survival rate of PDAC is approximately 10%,^1)^ whereas that of PanNETs is approximately 40%,^2)^ indicating a comparatively favorable prognosis. However, 21%–80% of patients present with metastatic disease at diagnosis, and 40%–93% of these patients have liver metastases.^3,4)^ The presence of liver metastases is associated with a poor prognosis in NET patients.^5)^ In particular, adrenocorticotropic hormone (ACTH)-producing tumors have unfavorable outcomes, with reported 5-year survival rates as low as 16%.^6)^ In addition to a poor prognosis, excessive ACTH secretion often leads to severe Cushing’s syndrome, including hypercortisolism-related metabolic and infectious complications that can be clinically problematic.^7)^ Therefore, the precise localization of hormone-producing lesions is essential for appropriate treatment planning. Surgical resection remains the only curative option for neuroendocrine liver metastases.^8)^ However, both pancreatectomy and major hepatectomy are highly invasive procedures, and simultaneous resection significantly increases the risk of postoperative complications. Pancreatoduodenectomy (PD) combined with major hepatectomy carries a 7.69-fold higher risk of postoperative infectious complications than hepatectomy alone.^9)^ For functional NETs, hormonal control is as important as tumor volume control, making treatment strategy decisions particularly critical.

To detect the lesion responsible for hormone secretion, selective arterial calcium injection (SACI) testing or selective arterial secretin injection (SASI) testing can be employed.^10,11)^

The SACI test is a functional diagnostic procedure primarily used for localizing insulinomas and gastrinomas. Calcium is selectively injected into the arteries supplying the pancreas, and hormone levels are measured in the hepatic veins to identify the responsible region.

Furthermore, we recently developed a method termed hormonal mapping, in which selective venous sampling is used to identify the hepatic regions responsible for hormone production. This approach facilitates treatment decisions that prioritize symptom alleviation in cases with multiple metastases where complete resection is not feasible because of an insufficient future liver remnant.^12)^

Here, we report a rare case of an ACTH-producing PanNET in the pancreatic head with multiple liver metastases, in which curative resection was achieved using a staged, minimally invasive approach guided by hormonal mapping.

## CASE PRESENTATION

A 42-year-old woman presented at a local hospital with weight gain, severe edema, and hypertension. Laboratory tests revealed hypokalemia, with a serum potassium level of 2.5 mEq/L. Elevated cortisol (19.4 μg/dL) and ACTH (136.0 pg/mL) levels raised suspicion of ACTH-dependent Cushing’s syndrome. A 1-mg dexamethasone suppression test demonstrated elevated urinary free cortisol and high serum ACTH levels, confirming the diagnosis.

Contrast-enhanced CT showed a solitary mass in the pancreatic head and five liver masses. A liver biopsy confirmed the diagnosis of NET G2 with a Ki-67 index of 6.2%. Cortisol synthesis was suppressed using metyrapone, and dexamethasone replacement therapy was initiated. To control the symptoms of cortisol excess, the patient was treated with oral metyrapone (2000 mg/day), which improved hypertension, hyperglycemia, electrolyte imbalance, and edema. Subsequently, the patient was referred to our hospital.

**Fig. 1:**
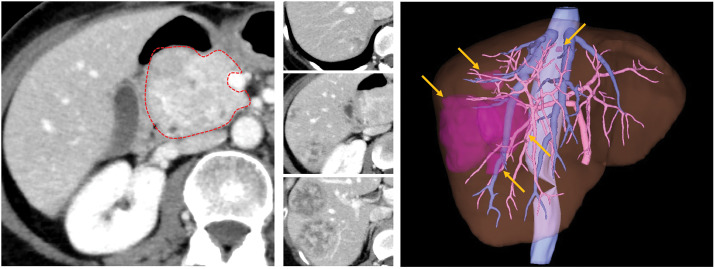
CT images of primary (red dotted line) and metastatic lesions (orange arrows) and 3D reconstructions by using SYNAPSE VINCENT (Fujifilm Healthcare Corporation, Tokyo, Japan). A primary lesion measuring 47 mm in diameter was observed in the head of the pancreas, showing contrast enhancement in the early phase. There were also 4 lesions in the posterior segment of the liver and 1 lesion in the root of the MHV. MHV, middle hepatic vein

**Fig. 2:**
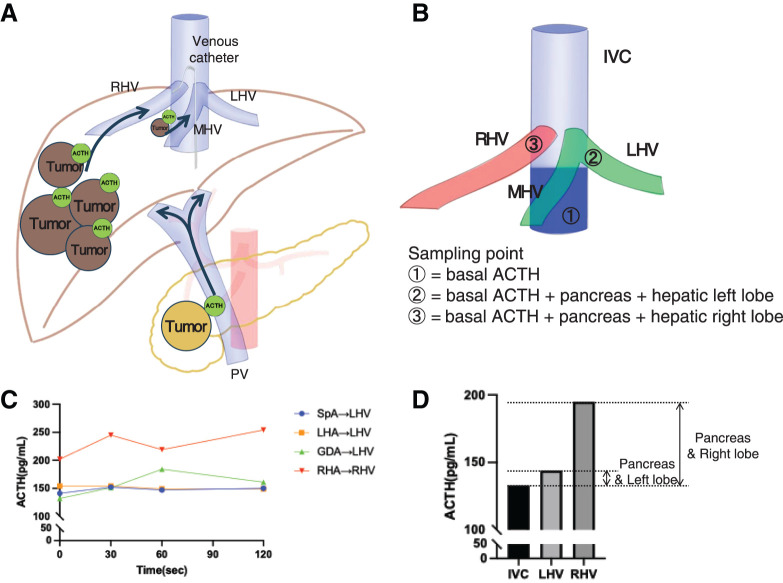
Localization of hormone-producing lesions by venous sampling. (**A**) Schematic illustration of the tumor location and potential sites of hormone production. (**B**) Venous sampling points and the corresponding anatomical regions reflected by each site. (**C**) Changes in ACTH levels after stimulation. ACTH levels increased approximately 1.4-fold with GDA stimulation and 1.2-fold with RHA stimulation; however, no distinct step-up was observed. (**D**) Comparison of basal ACTH levels among sampling sites, showing the highest concentration in the RHV, which suggested that the right hepatic lobe was the predominant source of ACTH production. ACTH, adrenocorticotropic hormone; GDA, gastroduodenal artery; IVC, inferior vena cava; LHA, left hepatic artery; LHV, left hepatic vein; MHV, middle hepatic vein; RHA, right hepatic artery; RHV, right hepatic vein; SpA, splenic artery

On re-evaluation at our institution, CT revealed a 47-mm hypervascular tumor in the pancreatic head and five liver metastases, with the largest measuring 54 mm. Four lesions were located in the posterior section and one lesion in segment 8 of the liver (**Fig. 1**). Initially, a one-stage curative resection was considered, which required PD plus extended posterior sectionectomy. A two-stage, minimally invasive approach was selected to ensure perioperative safety and reduce surgical invasiveness, including intraoperative blood loss. Therefore, we explored a staged resection strategy and performed hormonal mapping to identify the ACTH-secreting lesion. The procedure is shown in **Fig. 2**. Because most liver metastases were located in the posterior section, ACTH secretion was expected to be detectable in the right hepatic vein (RHV) (**Fig. 2A**). Portal vein sampling, although the most accurate method for assessing ACTH secretion from the pancreatic head, was considered technically demanding and potentially risky. Therefore, we assumed that ACTH secretion from pancreatic tumors would be evenly distributed across all hepatic veins. Accordingly, the inferior vena cava (IVC) blood below the hepatic vein confluence was considered the baseline; RHV samples represented ACTH secretion from the pancreas plus right lobe, and left hepatic vein (LHV) samples represented ACTH secretion from the pancreas plus left lobe (**Fig. 2B**). Calcium (3 mL of 8.5% calcium gluconate hydrate; CALCICOL Injection, Nihon Pharmaceutical, Tokyo, Japan) was sequentially injected into the splenic artery (SpA), left hepatic artery (LHA), gastroduodenal artery (GDA), and right hepatic artery (RHA). Venous blood was collected from the RHV during the RHA stimulation and from the LHV during other stimulations. The results are summarized in **Table 1** and **Fig. 2C**. RHV sampling revealed an ACTH level of 195 pg/mL, whereas IVC and LHV samples showed levels of 133 and 144 pg/mL, respectively (**Fig. 2D**). No significant increase was observed with arterial stimulation, suggesting that liver metastases were the predominant source of ACTH secretion.

**Table 1:** Baseline ACTH levels before arterial stimulation and ACTH changes with arterial stimulation

	Stimulation site	Collection site	Sec			
			0	30	60	120
ACTH (pg/mL)						
Venous sampling		IVC	133			
		LHV	144			
		RHV	195			
SACI test	SpA	LHV	141	152	147	150
	LHA	LHV	154	154	149	149
	GDA	LHV	132	151	184	161
	RHA	RHV	202	245	219	254

ACTH, adrenocorticotropic hormone; GDA, gastroduodenal artery; IVC, inferior vena cava; LHA, left hepatic artery; LHV, left hepatic vein; RHA, right hepatic artery; RHV, right hepatic vein; SACI, selective arterial calcium injection; Sec, seconds; SpA, splenic artery

**Fig. 3:**
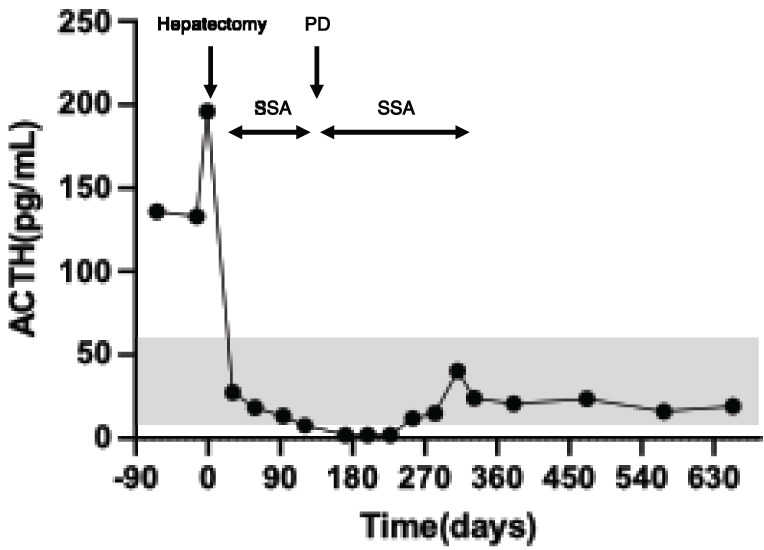
Temporal trend of ACTH levels. At the initial presentation, the ACTH level was elevated to 136 pg/mL. Following the 1st surgery for the right lobe lesions, the ACTH level returned to within the normal range. During the subsequent follow-up period with SSA therapy, the ACTH elevation did not recur. ACTH, adrenocorticotropic hormone; dACTH, adrenocorticotropic hormone; PD, pancreatoduodenectomy; SSA, somatostatin analog

**Fig. 4:**
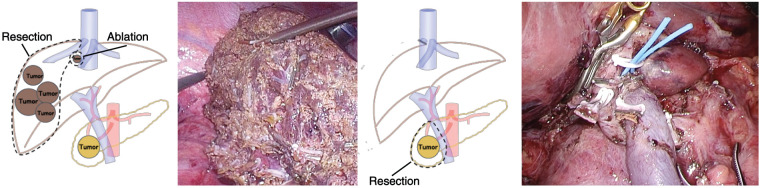
Staged laparoscopic hepatectomy and pancreaticoduodenectomy. The initial surgery consisted of a laparoscopic hepatectomy (1-L-675′-RHV) and microwave ablation for S8 (1 site). Three months later, a 2nd surgery—laparoscopic pancreaticoduodenectomy—was performed. RHV, right hepatic vein; S8, segment 8

**Fig. 5:**
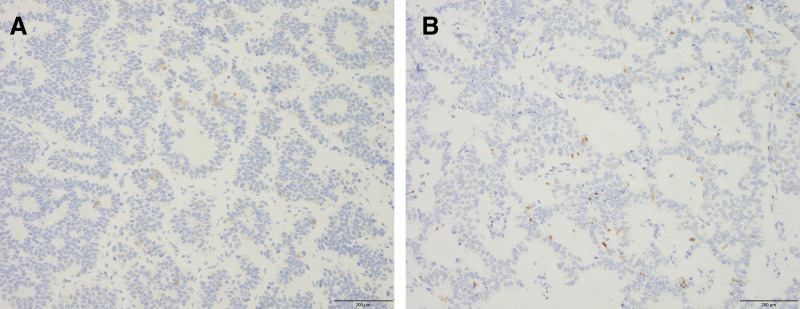
Immunohistochemical findings of the surgical specimens using anti-ACTH staining. (**A**) A pathological specimen from the liver and (**B**) the head of the pancreas. Both specimens showed only weak positive immunostaining for ACTH. ACTH, adrenocorticotropic hormone

Based on these findings, a laparoscopic posterior sectionectomy and microwave ablation of segment 8 were performed. The operative time was 8 h 42 min, and the blood loss was 150 mL. The patient was discharged 15 days later without any complications. On POD 31, ACTH normalized and the Cushingoid symptoms resolved (**Fig. 3**). Oral metyrapone was reduced from 2000 to 250 mg/day. Perioperative steroid replacement therapy was also administered. During the first hepatectomy, hydrocortisone sodium succinate (Solu-Cortef; Pfizer Japan, Tokyo, Japan) was administered at 300 mg/day on the day of surgery and tapered thereafter, reaching a maintenance dexamethasone dose (DECADRON; Nichi-Iko Pharmaceutical, Toyama, Japan) of 0.5 mg/day during metyrapone therapy. She was also treated with lanreotide, a long-acting somatostatin analog (SSA), as adjuvant therapy. Although there is no clear recommendation in the current Japanese guidelines regarding postoperative medical therapy after the resection of functional NETs, lanreotide was administered in this case because our institution routinely uses SSA in patients considered to be at high risk of new lesion development or recurrence. Follow-up contrast-enhanced CT (2 months) and gadoxetic acid–enhanced MRI (EOB-MRI) (3 months) showed no recurrence, and laparoscopic PD was performed (**Fig. 4**). Operative time was 10 h 28 min, with 125 mL of blood loss. R0 resection was achieved. Postoperatively, the patient developed Clavien–Dindo grade II delayed gastric emptying (DGE) but was discharged on day 30. For the second PD, hydrocortisone sodium succinate was administered at 200 mg/day and gradually tapered to oral hydrocortisone (Cortril; Pfizer Japan, Tokyo, Japan) at 10 mg/day at discharge, which was completely discontinued during outpatient follow-up. Pathology confirmed NET G2 in both specimens, with Ki-67 indices of 9.4% (liver) and 4.5% (pancreas). Immunohistochemistry revealed weak ACTH staining (**Fig. 5**).

At 21 months after the pancreatectomy, the patient remains alive and recurrence-free, and no longer requires metyrapone or SSA therapy.

## DISCUSSION

ACTH-producing neuroendocrine tumor (ACTHoma) is rare, and its management is challenging when accompanied by synchronous liver metastases.^13)^ When both pancreatic and hepatic lesions are present, simultaneous PD and liver resection may be required, which carries a high risk of severe complications.^9)^ Liver resection is prioritized for surgical safety, as choledochojejunostomy carries a high risk of retrograde infection.^14)^ In contrast, functional NETs often require resection of hormone-producing lesions for symptom control.^7)^ Because of tumor heterogeneity,^15)^ the hormone-producing site is not always clearly identifiable, making localization through hormonal mapping essential for planning the optimal surgical strategy.^12)^

In this case, hormonal mapping was performed to identify the dominant ACTH-producing site. While the SACI test is well established for insulinomas and gastrinomas, where increases of over 3-fold and 1.2-fold from baseline, respectively, are considered diagnostic, no standardized cutoff exists for ACTH-producing tumors.^16,17)^ In this patient, the ACTH response to arterial stimulation was modest (1.2–1.4-fold), but baseline RHV levels were higher than IVC and LHV levels, suggesting right hepatic lobe predominance. This was validated by normalization of ACTH levels and resolution of Cushingoid features after right lobe resection. Mori et al. diagnosed an ACTH-producing pancreatic NET based on a 1.6-fold increase in ACTH levels during the SACI test.^18)^ However, we estimated the hormone-producing site from venous sampling results, and surgical resection confirmed this, as evidenced by postoperative normalization of ACTH levels. Interestingly, hormone expression assessed by immunohistochemical staining of tissues does not necessarily correlate with actual hormone production^19)^ In this case, both liver and pancreatic specimens showed weak ACTH staining, unreflective of actual hormone secretion. This finding underscores the limitation of histopathology in hormone-producing tumors and the importance of functional diagnostic tests such as the SACI test.

We acknowledge some limitations. Although portal vein sampling provides the most accurate assessment of pancreatic hormone secretion, it is highly invasive; we compared ACTH levels between the IVC and LHV as substitution. ACTH levels in the IVC and LHV were comparable, while those in the RHV were elevated. This, together with postoperative hormonal normalization, verified the right hepatic lobe as the main source of ACTH. Additionally, evaluation of the segment 8 lesion may have been limited by the venous sampling approach. This limitation should be considered when interpreting the hormonal mapping results. Adopting a staged resection strategy allowed sufficient recovery after hepatectomy before PD, reducing complication risk. Recurrence rates after hepatic resection for NET metastases range from 46% to 64% within five years,^20,21)^ with 70% occurring within three years. However, no standardized intervals exist for staged resections. In this case, the second surgery was performed three months after hepatectomy, based on the patient’s rapid recovery and stable hormonal control, with close surveillance for early recurrence during the interval.

By performing liver resection first, guided by hormonal mapping, we reduced these risks and achieved safe and potentially curative outcomes.

## CONCLUSIONS

Hormonal mapping with selective venous sampling enabled the precise localization and safe staged resection of an ACTH-producing pancreatic NET with synchronous liver metastases, demonstrating its clinical value in guiding tailored surgical strategies for functional NETs.

## DECLARATIONS

### Availability of data and materials

The data supporting the findings of this study are available from the corresponding author upon reasonable request.
